# Short-Term PM2.5 Forecasting Using Exponential Smoothing Method: A Comparative Analysis

**DOI:** 10.3390/s18103223

**Published:** 2018-09-25

**Authors:** Sachit Mahajan, Ling-Jyh Chen, Tzu-Chieh Tsai

**Affiliations:** 1 Institute of Information Science, Academia Sinica, Nangang District, Taipei City 115, Taiwan; cclljj@iis.sinica.edu.tw; 2 Social Networks and Human-Centered Computing, Taiwan International Graduate Program, Academia Sinica, Nangang District, Taipei City 115, Taiwan; 3 Department of Computer Science, National Chengchi University, Wenshan District, Taipei City 116, Taiwan; ttsai@cs.nccu.edu.tw

**Keywords:** Internet of Things, air quality forecast, PM2.5, Smart Cities

## Abstract

Air pollution is a global problem and can be perceived as a modern-day curse. One way of dealing with it is by finding economical ways to monitor and forecast air quality. Accurately monitoring and forecasting fine particulate matter (PM2.5) concentrations is a challenging prediction task but Internet of Things (IoT) can help in developing economical and agile ways to design such systems. In this paper, we use a historical data-based approach to perform PM2.5 forecasting. A forecasting method is developed which uses exponential smoothing with drift. Experiments and evaluation were performed using the real-time PM2.5 data obtained from large scale deployment of IoT devices in Taichung region in Taiwan. We used the data from 132 monitoring stations to evaluate our model’s performance. A comparison of prediction accuracy and computation time between the proposed model and three widely used forecasting models was done. The results suggest that our method can perform PM2.5 forecast for 132 monitoring stations with error as low as 0.16 μg/$m^{3}$ and also with an acceptable computation time of 30 s. Further evaluation was done by forecasting PM2.5 for next 3 h. The results show that 90 % of the monitoring stations have error under 1.5 μg/$m^{3}$ which is significantly low.

## 1. Introduction

With the rapid urbanization and industrial growth, the concern about deteriorating air quality is also increasing. Deteriorating air quality has adversely influenced the quality of life and even has affected the economic growth in a negative way. While there is still lot to do to solve this problem, IoT technology has come as a glimmer of hope. The idea is to use IoT devices and cognitive computing to generate large amount of data which can be further used to enhance air quality management systems and forecasting. A typical case would include the collection and storage of data obtained from the sensors, data analytics, prediction, visualization and an alert message service in the case of unusual behavior in the air quality. When talking about Smart City Initiative, an important part of it includes developing a system not only to monitor the air quality but also provide a future forecast. Among all the pollutants, PM2.5 (fine particulate matter with diameter less than 2.5 micrometers) are considered to be very harmful for humans. These particles are responsible for causing serious respiratory diseases, asthma, and lung cancer [1] as they can penetrate into the alveolus (regions for gas exchange in lungs).

Some previous research works have already shown how participatory sensing can be utilized for event detection [2]. In addition, wireless sensor networks can be efficiently implemented for real-time monitoring of environment [3]. However accurately predicting air quality is very challenging task. Many different factors affect a city’s air quality, such as meteorological factors and traffic density [4]. Because of the nature of these factors, it is difficult to get data that are accurate enough to do the task. It is important to develop air quality forecasting systems that can produce accurate predictions with limited parameters. Most of the works done in this area rely on mathematical models. The simulations are run to do the forecasting [5]. Many approaches have been proposed for air quality management (AQM). These can be categorized into the following: empirical model based prediction, fuzzy logic based modeling, simulation based and statistical model based [6]. Having an air pollutants monitoring system is of utmost importance to efficiently monitor the changes in the air quality and assess the harmful impact of air pollution on human health and sustainability of cities. Some works [7,8] discuss air quality monitoring for indoor and outdoor locations. Another approach is to use air quality modeling software. The drawback of using such models is that they do not consider all the factors that can affect air quality. At present, there are a few organizations which have platforms for monitoring and understanding air quality (https://www.londonair.org.uk/LondonAir/Default.aspx). Most of them follow the conventional approach of collecting data, analysis and repeating the whole process again. It has to be realized that there is always a cost associated with data collection and replicating the data is a tedious job. This makes it important to have a cost-effective and a reliable system. Our method is data-centric. We used PM2.5 data obtained from a large scale IoT deployment in Taiwan [9]. We used the real-time data to perform the experiments and evaluation.

The contribution of this paper is four-fold:

- A univariate time series prediction model is developed that performs forecast using exponential smoothing with drift.
- The proposed model was used for hourly PM2.5 prediction using real-world data obtained from monitoring devices deployed in Taiwan.
- We evaluated our model’s performance by comparing the results with three baseline models by using data from the monitoring stations. The evaluation was based on accuracy and computation time. The model was further tested for forecasting PM2.5 for the next three hours.
- The scalability of the model is tested by performing forecast for 132 air quality monitoring nodes deployed in Taichung region in Taiwan.

The rest of the paper is organized as follows. In Section 2, we discuss the related works and our motivation behind this study. In Section 3, we describe the system overview which includes the proposed architecture, Airbox Project and the deployment. We also discuss the Airbox Data and the visualization platforms. In Section 4, we explain the proposed model in detail and also discuss other baseline models. In Section 5, we implement the model on real time data and observe the results. In Section 6, we evaluate our model by comparing the results of the proposed model with other baseline models. Section 7 concludes the paper and gives a short description about the possible future works.

## 2. Related Work and Motivation

Much effort has been made by utilizing the modern day technology to develop systems which can provide real-time information and services to the users. There have several works related to air quality monitoring and providing services to the users. Grover et al. [10] proposed a Deep Hybrid Model for weather forecasting. It does not forecast PM2.5 but it predicts variables such as temperature, wind and dew point. Linear regression technique has been widely used for forecasting and analysis, including data pertaining to financial domain [11], which is fast-changing, meteorology, environment data [12], etc. However, due to complex nature of air quality data, it is not extensively used in this domain. Zheng et al. [4] performed forecasting over the next 48 h using a data driven approach. They used a predictive model based on a linear regression model and neural network. Kitchin [13] proposed that big data can enable real-time analysis of cities, urban governance and can be used as an effective tool to develop smart and sustainable cities. Time-series data can be noisy in many cases and it is not easy to perform forecast with non-stationary data. Ghazali et al. [14] used a Dynamic Ridge Polynomial Neural Network for financial time-series prediction using higher order and recurrent neural network. Hsieh et al. [15] focused on issues such as air quality forecast for an area using data from sparse monitoring stations. Khan et al. [16] investigated the use of cloud platform for big data analytics. They demonstrated how the smart city initiative can be realized by using real-time big data stored in cloud platforms. However, the loopholes of these techniques are that they simply rely on feeding a variety of features to the model. Those features belong to one particular location and a similar model is used for all the locations. The problem is that every location has different emission sources, pollutants, and concentrations; therefore, one model cannot perform accurate forecasting for all locations. Donnelly et al. [17] proposed a linear regression based method for real-time air quality forecast with high accuracy. The model is then tested for three urban sites and one rural site. In our work, we made a scalable model and, to test the scalability of our system, we applied it to 132 monitoring stations. Zheng et al. [18] implemented machine learning techniques and big data to perform urban computing. To avoid too complicated computing, Zhu et al. [19] categorized air pollution before doing prediction. However, it may lose the meaning of time-series prediction in this way. Although machine learning models have been proposed widely, when it comes to PM2.5 prediction for small time intervals, it has not been exploited much. Machine learning techniques sometimes have computational efficiency issues. To tackle this problem, Shi et al. [20] and Chen et al. [21] used Xgboost which implements read-ahead caching and utilizes parallel computing to reduce the execution time. However, this might lead to decrease in the accuracy of the prediction model.

Humans are always surrounded by sensing devices which create a kind of fusion between physical world and virtual world. In another way, we can term it as Internet of Humans (IoH) which is a combination of (IoT) and Human-Centered Computing (HCC). Regular air quality monitoring and analysis can help in improving community awareness on environmental issues. In this work, we combine networked sensing and crowd-sourcing techniques to collect streams of sensing data about the surroundings and provide insightful information service at personal, society and urban levels. Our motivation behind this work was to integrate IoT and machine learning techniques and develop a system that not only provides real-time air quality information to the users but also creates awareness among the people about issues related to poor air quality. In this work, we deal with large amount of PM2.5 data obtained from the IoT devices deployed around Taiwan. Thus, it becomes very challenging to make sure that the data is of highest quality and any anomaly in the data is detected. In addition, it becomes important to achieve high prediction accuracy and also make sure that the computation time is low so that the model can be used to create a real-time application.

## 3. System Overview

### 3.1. Proposed System Architecture

**Figure 1:**
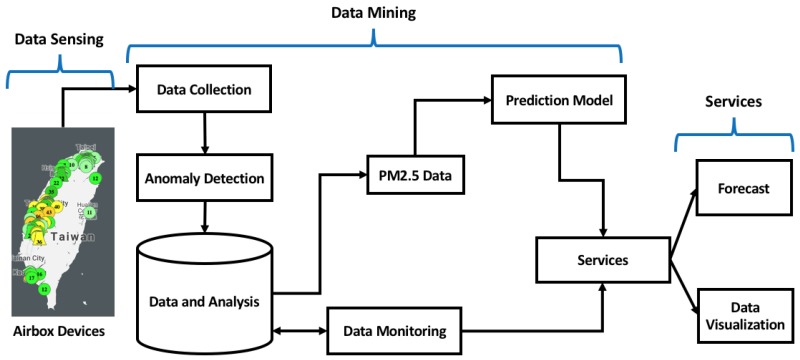
Architecture of the proposed system.

The proposed system is shown in Figure 1. It follows a three-way approach which includes data sensing, data mining and providing services. The data were obtained from the PM2.5 sensing devices which are deployed all over Taiwan. The IoT devices provide real-time PM2.5 data, temperature and humidity levels for different regions. The data collection was a continuous process and any anomaly in that process was directly reported to the administrator [22]. The collected data were stored in the database and easily accessible. Sometimes some of the monitoring stations do not show any readings. Data monitoring component takes care of it by filtering such data and filling in the missing values depending on spatial and temporal neighboring devices. The forecast model uses exponential smoothing with a drift to predict hourly PM2.5. The forecast data can be stored and provided as a web-service side by side with the visualization of the data.

### 3.2. Airbox Project

**Figure 2:**
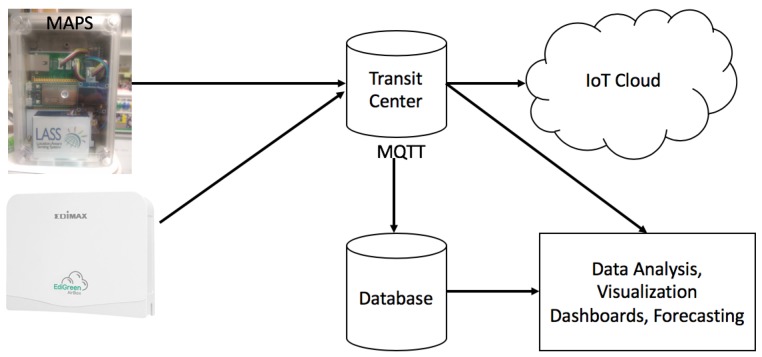
Flowchart for PM2.5 Sensing Framework.

The Airbox Project comprises of pilot deployment of IoT systems for PM2.5 monitoring all over Taiwan. The main motive of this project is to encourage people and motivate them to participate in PM2.5 sensing. The main inspiration behind this project is the LASS (Location Aware Sensing System) community. This community engages the people to participate in PM2.5 sensing and also encourages them to try and develop sensing devices by themselves. The project facilitates PM2.5 monitoring at a finer spatiotemporal granularity and enriches data analysis by making sure that all the measurement data are available freely to everyone [9]. The devices are installed in buildings with reliable Internet connection and power source. In addition, the data (https://pm25.lass-net.org/en/) are easily accessible which makes data analysis easy. The sensing devices in Airbox Project are designed and developed by professional manufacturers. The industrial product level devices are made in close cooperation with Edimax Inc. and Realtek Inc. in Taiwan. The devices are based on Realtek Ameba development board. The device contains a PMS5003 PM2.5 sensor and a HTS221 temperature/humidity sensor. Another version of deployed device is called MAPS (Micro Air Pollution Sensing System) which is developed by Network Research Lab at Institute of Information Science, Academia Sinica, Taiwan. It is based on MediaTek LinkIt Smart 7688 Duo development board. It has a PMS5003 sensor for PM2.5 and BME 280 for temperature/humidity. The data sensing part of the framework is shown in Figure 2. There are three major components of the sensing system.

1. *Data Producers* comprise the sensors which provide sensed data. The hardware and the source codes are open source so that people can create such devices themselves.
2. *Transit Centers* act as data brokers for the data sent from the data producer to data users. Multiple data brokers can be used to achieve scalability and fault tolerance.
3. *Data Users* are those who use the sensed data, analyze it and create different types of applications.

**Figure 3:**
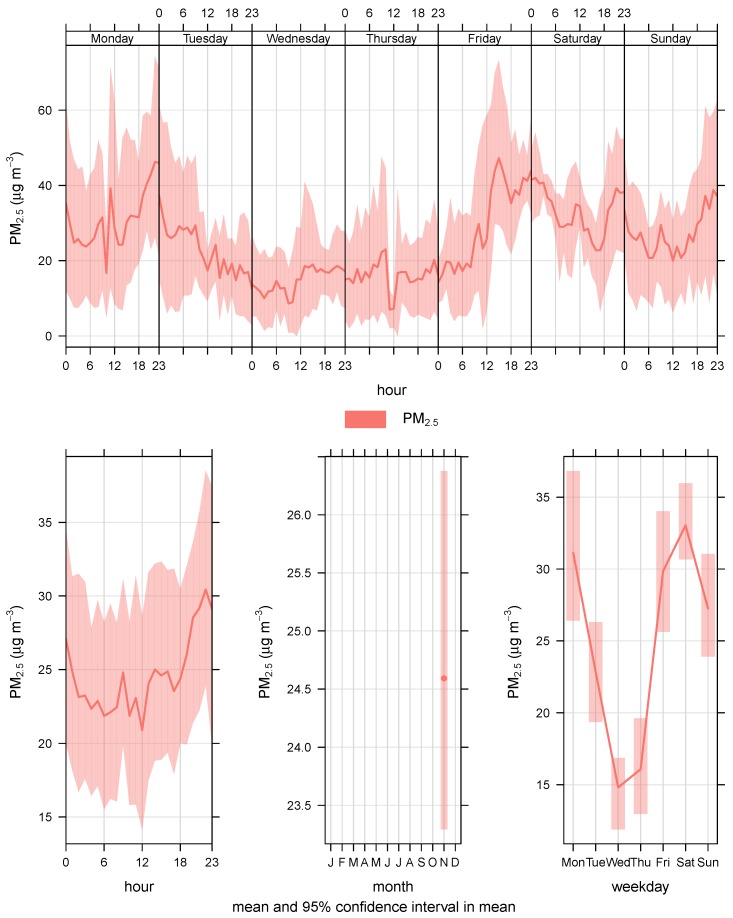
Hourly and daily PM2.5 variations.

**Figure 4:**
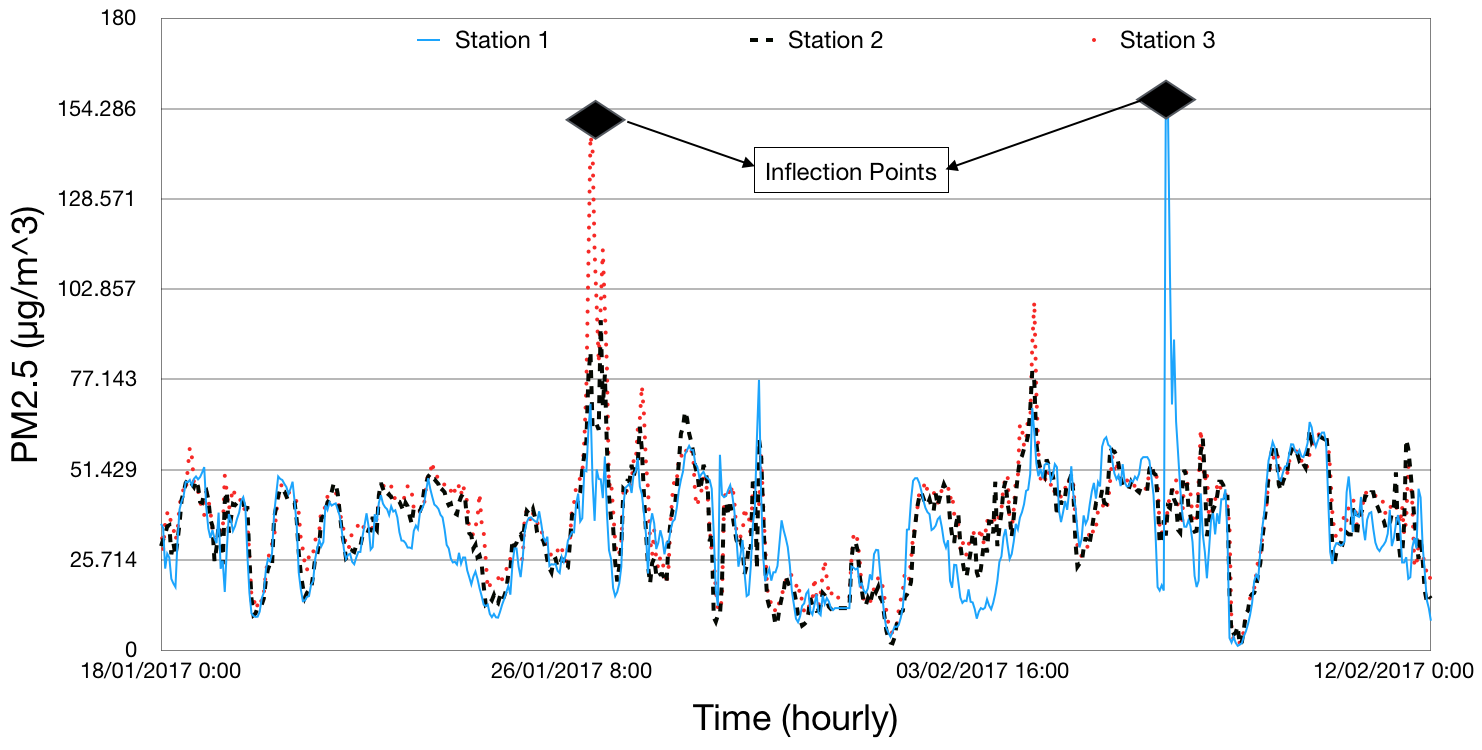
Hourly representation of PM2.5 μg/$m^{3}$ for three stations.

For data communication, the sensing framework uses Message Queuing Telemetry Transport (MQTT) protocol [23]. MQTT is used because of it lower communication overhead, simple design and flexibility to adjust to different formats of messages. Data sampling frequency for the Airbox devices is estimated to be every 5 min. However, it was observed that the inter-sample time was 6 min for almost 80% of the devices and for the remaining it was around 12 min. There is a standby time between sample collection of an Airbox device and is found to be 5 min and it takes about 1 min to do the sampling. This makes the inter-sampling time to be 6 min. First data measurement fails in the case there is an error. In such cases, the inter-sampling time increases to 12 min. For this research, we converted the data into hourly data. The PM2.5 data were checked for missing data before proceeding with the experiments. Figure 3 shows the PM2.5 variations for based on data for the month of November 2016. It can be observed in Figure 3 that sometimes there is a trend in PM2.5 variations. For example, during the weekends, it can be assumed that most people would go out, which means more traffic and more pollutants. Thus, higher PM2.5 would be observed during the weekends rather than the weekdays. Similarly, during the morning and late evening, PM2.5 would be higher as people would be commuting. Such trends are easy to observe. In Figure 4, it can be observed that hourly PM2.5 concentrations shows different levels for different stations. Variations can be seen for different stations at different time periods. The peaks in the plot can be referred to as inflection points, i.e., the points at which the PM2.5 concentration level changes sharply. Inflection points can be considered as sudden increase in the PM2.5 values which might be caused by environmental factors or human activities. At these points, the PM2.5 concentration level changes sharply. These variations do not represent the regular air quality pattern at a particular location but an incident which might happen because to thunderstorms or strong winds. As these incidents are rare, so it is very difficult to model them using a conventional forecast model.

### 3.3. Data Archive and Open Data API

A data archive service has been setup which stores and provides all the records from the monitoring devices. Having such a system is very beneficial as such a service ensures that all the PM2.5 observations based on on our deployment can be easily accessed and traced. In addition, it helps us to maintain a data archive that is long lasting and contains verified data for further analysis and modeling. Another important feature of this system is that it can import PM2.5 measurement data from other open local data sources in Taiwan. This actually helps to improve the coverage area and get more and more data. Through the open data API (in the JSON data format), people can access the latest measurement data of a particular AirBox device, leading to thousands of data for any device on any particular date.

### 3.4. Visualization Platforms for Airbox Data

**Figure 5:**
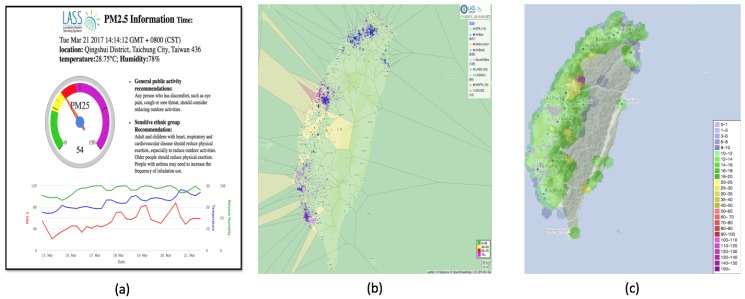
Data Visualization Platforms: (**a**) device status; (**b**) Voronoi diagram; and (**c**) real-time PM2.5 visualization.

**Figure 6:**
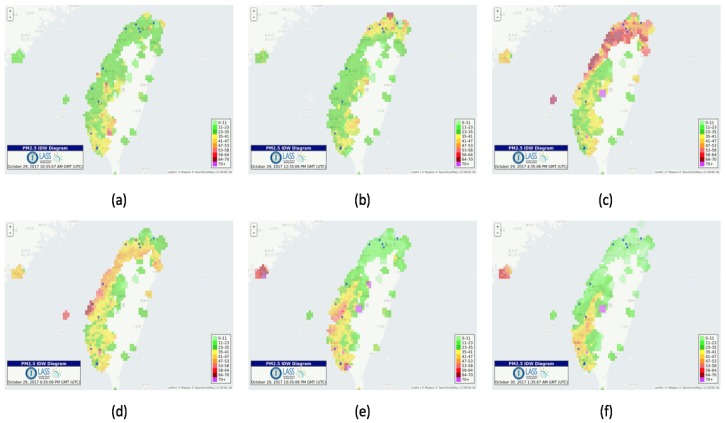
(**a**–**f**)—IDW animation for a pollution incident.

Visualization platforms have been developed to visualize the Airbox data. One of them is a visualization system that gives information about every Airbox device. These services help in understanding the impact of spatiotemporal factors on PM2.5 measurement. A dashboard has been setup that helps in visualizing the device data over a period of time. The dashboard shows PM2.5, temperature and relative humidity as shown in Figure 5a. A Voronoi diagram and real-time PM2.5 monitoring visualization has also been developed which is updated every 5 min, as shown in Figure 5b,c. A Voronoi diagram represents a partitioned plane into regions based on the distance to a specific subset. In our case, it is this the sensor location. An animation application has been developed which shows the air quality for last 24 h in the form of IDW (Inverse Distance Weighting) animation. The animation is available for whole Taiwan as well as all major regions including Taipei, Taoyuan, Taichung and Tainan. The animation is updated every 1 h. There have been many cases in which sudden change in air quality is noticed. Such animations help in understanding the trend followed by the air pollution. For major air pollution incidents, the results are regularly shared online and can also be used by the policy making agencies to analyze the trend. Figure 6 gives an example of how the IDW animation can help in understanding the trend in air pollution dispersion. It can be observed that, in the initial frame, the PM2.5 levels are normal. The air quality starts deteriorating in the northern part of Taiwan as see in the second frame. Soon, it covers the whole northern region and starts dispersing towards northwestern Taiwan. It further disperses towards the central and southern Taiwan and it can be observed that pollutants intensity decreases over the time.

## 4. Methodology

In this section, we discuss in detail the framework of the prediction model. We also discuss the other three baseline models that were used to perform the comparative analysis. The models used for performing the analysis are some of most widely used time-series forecasting models: Autoregression Integrated Moving Average (ARIMA), Neural Network Autoregression Model (NNAR) and Hybrid Model [24].

### 4.1. Forecasting Method Using Exponential Smoothing with Drift (ESD) Model

**Figure 7:**
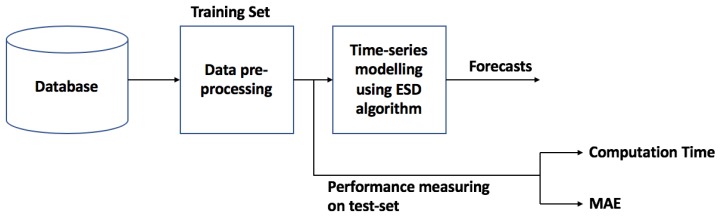
ESD modeling and forecasting framework.

Figure 7 shows the proposed ESD modeling and forecasting framework. The proposed method is based on the Theta method [25]. The idea behind using this method for forecasting is that when considering short-term PM2.5, we assume that there is no seasonality or trend. This method uses weighted moving average of the past data as the basis to perform the forecast. The theta line can be described as

(1) $$\nabla^{2}Z_{t}\left(\theta \right)=\theta \nabla^{2}Y_{t},t=3,\ldots ,n,$$

where $Y_{1}$…,$Y_{n}$ represents the non-seasonal original time-series and ∇ represents the difference operator. $Z_{1}$ and $Z_{2}$ can be obtained by minimizing $\sum_{t=1}^{n}{\left[Y_{t}-Z_{t}\left(\theta \right)\right]}^{2}$. Another analytical solution wisas proposed in [26]. It is given by

(2) $$Z_{t}\left(\theta \right)=\theta Y_{t}+\left(1-\theta \right)\left(A_{n}+B_{n}t\right),t=1,\ldots ‥,n,$$

In Equation (2), $A_{n}$ and $B_{n}$ represent the minimum square coefficient of a linear regression over $Y_{1}$, $Y_{2}$, …, $Y_{n}$ against 1,‥‥*n*. The linear regression is denoted by

(3) $$A_{n}=\frac{1}{n}\sum_{t=1}^{n}Y_{t}-\frac{n+1}{2}B_{n}$$

(4) $$B_{n}=\frac{6}{n^{2}-1}\left(\frac{2}{n}\sum_{t=1}^{n}tY_{t}-\frac{1+n}{n}\sum_{t=1}^{n}Y_{t}\right)$$

Based on these, it can be inferred that theta lines can be considered linear regression model’s functions applied directly to data.

The Theta method can be simplified as a case of simple exponential smoothing with a drift term which is equal to half the slope of a straight line fitted to the data [26]. In simple form, the ESD model can be explained as:

(5) $$l_{t}=l_{t-1}+b+\alpha \varepsilon_{t}$$

(6) $$\hat{X_{t}}\left(h\right)=l_{t}+hb$$

In the above equations, *l* denotes the level and *b* stands for the drift. The *h* step forecast is denoted by $\hat{X_{t}}\left(h\right)$. α is the smoothing parameter and its value is always between 0 and 1. Weighted averages are used to calculate forecasts. The weights decrease exponentially and this is controlled by parameter α. $\varepsilon_{t}$ represents the one-step forecast error at *t* within-sample.

### 4.2. Baseline Models for Comparison

In this section, we discuss about the models that were used to perform the comparative analysis.

#### 4.2.1. Autoregressive Integrated Moving Average (ARIMA) Model

An ARIMA model is considered to be a robust model [27] when it comes to time-series forecasting. During the forecasting process, first the model is identified, estimation of parameters is done and then a diagnostic check is performed. An ARIMA (*p*,*d*,*q*) model consists of *p*, *d* and *q* which are integers. They should be greater than or equal to zero and point to the order of the autoregressive (AR), integrated (I) and moving average (MA) components of the model [27]. Let us consider a time-series $Z_{t}$, where *t* is an integer and $Z_{t}$ denotes real numbers which correspond of values at a given time *t*. An ARIMA (*p*,*d*,*q*) model can be denoted by the following equation.

(7) $${\left(1-B_{s}\right)}^{d}\left(1-\sum_{i=1}^{p}\phi_{l}B_{s}^{l}\right)Z_{t}=\left(1+\sum_{i=1}^{p}\theta_{l}B_{s}^{l}\right)\varepsilon_{t}$$

In Equation (7), $B_{s}$ denotes the backward shift operator, and $\phi_{l}$ and $\theta_{l}$ are the autoregressive and moving part parameters. $\varepsilon_{t}$ is the error term. If *d* = 0, then it becomes an ARMA model [28].

#### 4.2.2. Neural Network Autoregression (NNAR) model

Lately, Artificial Neural Networks (ANN) have been used extensively when it comes to time-series forecasting. ANNs can be used to model the complicated relationships between input and output variables. When we talk about NNAR model, the input is a lagged time-series and the output is predicted value of the time-series and is represented as NNAR (*p*,*P*,*k*)*m*. In the model, *p* and *P* indicate the lagged seasonal and non-seasonal inputs while *k* denotes the number of hidden layer nodes. *m* indicates the seasonality. The model has two functions. One is a linear combination function and the other one is an activation function. The linear combination function is denoted as

(8) $$Z_{t}=b+\sum_{i=1}^{n}w_{i,t}y_{t}$$

where $w_{i,t}$ represents the weight function, *b* represents the bias and $y_{t}$ are the lagged time series values. The weights are randomly selected initially and later they can be updated using a “learning algorithm” [29] that minimizes the cost function. The activation function can be denoted as

(9) $$f\left(Z_{t}\right)=1/\left(1+e^{-Z_{t}}\right)$$

In this work, we considered a feed-forward neural network which is based on the Nonlinear Autoregressive Model for time series forecasting.

#### 4.2.3. Hybrid Model

**Figure 8:**
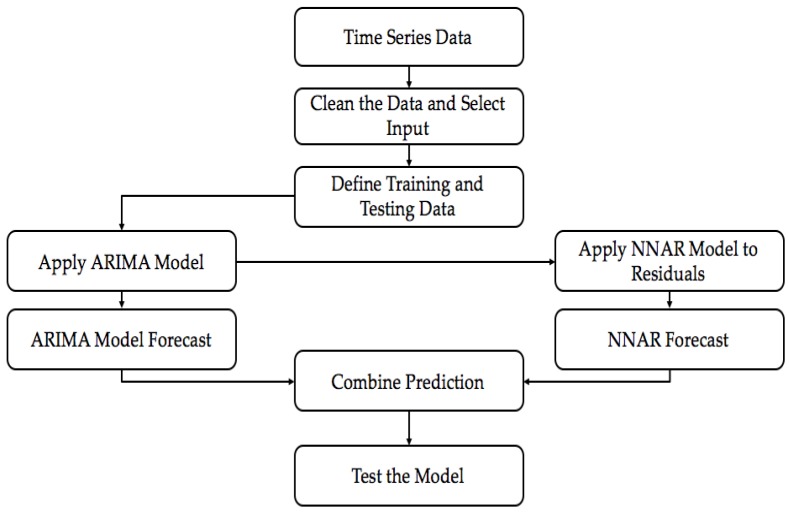
Flowchart for the Hybrid Model.

A time-series can be easily divided into linear and non-linear components. ARIMA model provides good forecasting but it cannot capture the non-linear components. This makes it important to have a technique which can capture the non-linear components too. This is when we can use ANNs. They can take care of the non-linear components of the data. Figure 8 shows the flowchart for the model. We used a Hybrid model [24] represented in Equation (10) where $X_{t}$ represents the linear components and $Y_{t}$ represents the non linear components.

(10) $$Z_{t}=X_{t}+Y_{t}$$

In the initial step, these two components have to be estimated from the data. Then, the next stage is the application of ARIMA model. At this stage, ARIMA model takes care of the linear components and the residuals in the form of non-linear components are generated. Let us assume that $R_{t}$ are the residuals generated at time *t* from the linear model. Thus, it can be written as

(11) $$R_{t}=Z_{t}-F_{t}$$

where $F_{t}$ is the forecast value for time *t*. These residuals are then modeled using neural networks. If we assume that there are *n* input nodes, then the neural network model for residuals can be given as

(12) $$R_{t}=f\left(R_{t-1},R_{t-2},\ldots ‥,R_{t-n}\right)+e$$

Neural network defines the non-linear function *f*, and *e* is the random error generated. Finally, the forecast from the neural network is generated and Equation (10) is used to get the final output. We used an ARIMA (3,1,1) model where 3,1,1 are the values of *p*, *d* and *q*, respectively. For neural networks, we used an NNAR(9,5,1) model which used nine lagged inputs with five nodes in the hidden layer. The parameters for ARIMA and NNAR model were chosen after testing different combinations and selecting the one which gave the best output. For the Hybrid model, we assigned equal weights to the prediction results from ARIMA and NNAR models.

## 5. Results

For this study, the measurement data were collected from the Airbox Devices installed in Taichung area of Taiwan. The measurement data were taken for the time period between 18 January 2017 and 12 February 2017. Most of the Airbox devices are installed in elementary schools around the region with regular power connection and internet supply. This makes the data very reliable and of better quality. Thus, to make the forecasting accurate, we only considered the stations with reliable data. To test the model, we considered the hourly PM2.5 data obtained from the monitoring stations deployed in Taiwan. Eighty percent of the data were used for training and 20% for testing the model. Mean Absolute Error (MAE) and computation time were used as the parameters to analyze the results.

If $y_{m}$ is the actual value and $\hat{y_{m}}$ is the predicted value, then the MAE can be denoted as

(13) $$\mathrm{MAE}=1/n\sum_{i=1}^{n}\left|y_{m}-\hat{y_{m}}\right|$$

**Figure 9:**
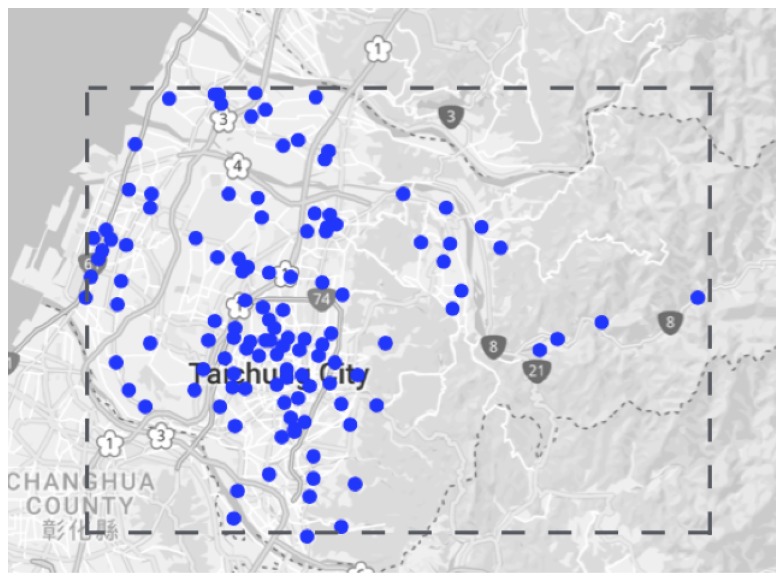
Geographical location of 132 monitoring modes.

**Figure 10:**
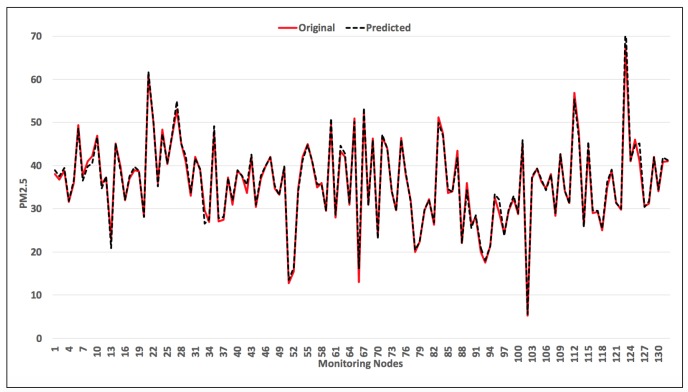
Comparison of observed and predicted PM2.5 values for 132 monitoring stations.

To check how our model performs the prediction, we did an hourly forecast for the 132 monitoring nodes. The geographical locations of the monitoring nodes is shown in Figure 9. Figure 10 shows a comparison plot between the observed and the predicted PM2.5 values. It can be observed that the predicted results are very close to the original observed results. This gives an idea about the scalability of the model when implemented on a large scale.

## 6. Evaluation

To evaluate, we followed a two-step process. In the first step, we compared our model with other baseline models to see how our model performs in comparison with the others. The parameters used were mean error and computation time. In the second step, we performed PM2.5 forecast for the next 3 h to see how the model deals with short-term PM2.5 forecasting.

### 6.1. Evaluation by Performing a Comparative Analysis with the Baseline Models

**Figure 11:**
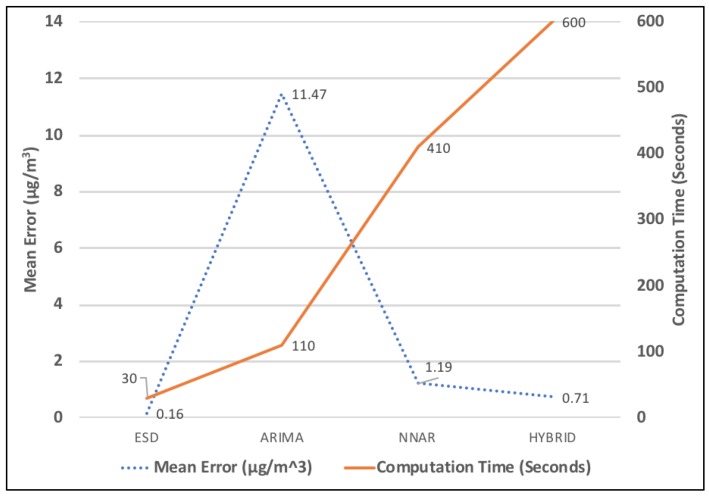
Mean error and computation time comparison with baseline models.

In this part, we compared the proposed model’s results with three baseline models. For comparison, we used ARIMA model, NNAR model and Hybrid model. These models are very well known models for forecasting time-series data. From the comparative analysis of all four models, as shown in Table 1, it can be observed that the ESD Model outperforms the other three models. The mean error obtained is 0.16 μg/$m^{3}$ which is significantly low when compared with other baseline models. Here, we also want to focus on the time–accuracy trade-off which can be observed in Figure 11. When talking about real-time applications, we do not only focus on low computation time but also on high accuracy. The ESD model satisfies both the conditions, i.e., high accuracy and low computation time.

**Figure 12:**
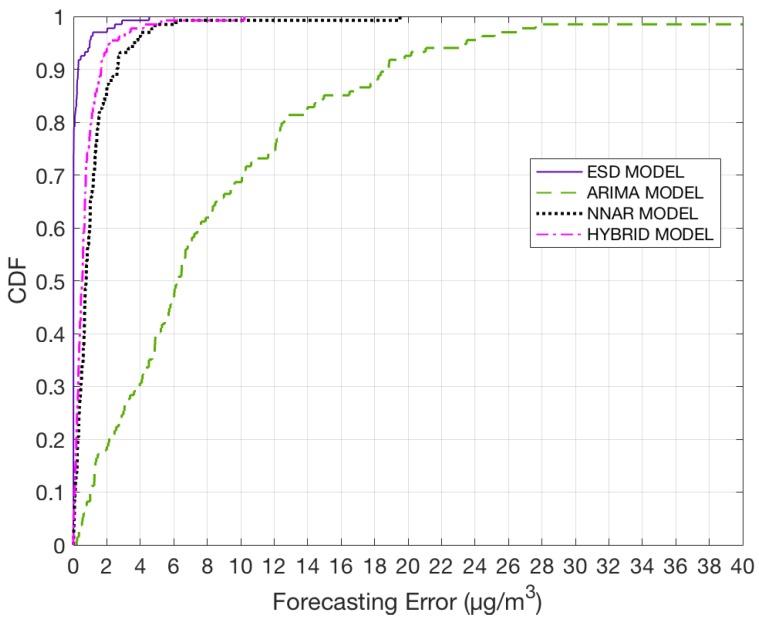
CDF plot for all four models for one hour prediction.

Figure 12 shows a cumulative distribution function (CDF) plot for all four models. It can be observed from the plot that the ESD model outperforms the other three models when performing prediction for the next hour. Around 95% of the monitoring stations show a forecasting error below 1 μg/$m^{3}$. Only NNAR and Hybrid model’s performance is close to the ESD model’s performance. However, it has to be taken into consideration that the maximum error calculated for ESD model is around 4 μg/$m^{3}$, whereas for Hybrid model it is around 10 μg/$m^{3}$ and for NNAR model it is around 19 μg/$m^{3}$.

### 6.2. Next 3 h PM2.5 Forecast Using the ESD Model

**Figure 13:**
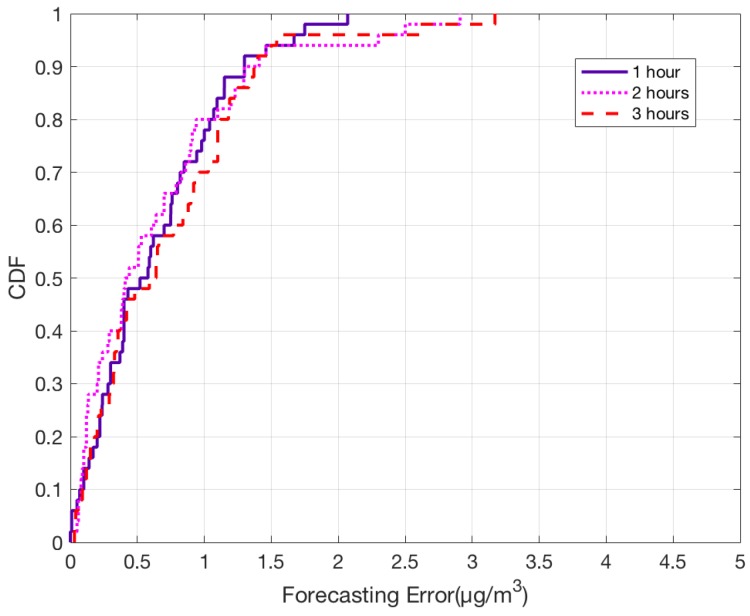
CDF plot for error between observed and predicted PM2.5 values for next 3 h.

Based on the results for one-hour forecast, we were able to understand that the system works well even when it is implemented on large number of monitoring stations. To further evaluate our model, we tested by performing PM2.5 forecast for next three hours for all the monitoring nodes. From the CDF plot shown in Figure 13, it can be observed that the forecasting error for most of the stations is significantly low. Almost 90% of the stations have error under 1.5 μg/$m^{3}$ for all cases. With these results, we can demonstrate that the proposed model can perform short-term PM2.5 prediction with high accuracy.

## 7. Conclusions and Future Works

As air pollution continues to affect the quality of life, there is a need to have a framework that would not only monitor the air quality but would also perform data analysis, air quality forecast and provide visualization services. To make sure that people know about the future air quality well in advance, it is important to come up with an accurate forecast system. In this paper, we integrated IoT technology and artificial intelligence to come up with a PM2.5 forecast system. We designed a forecasting model using exponential smoothing which performs hourly PM2.5 forecast based on real-time data obtained from the IoT devices deployed all over Taiwan. Parameters such as mean error, accuracy and computation time were used to analyze the results. To evaluate, we tested the model on 132 monitoring stations. We compared the ESD model’s results with three baseline models. With ESD model, we were able to obtain an mean error as low as 0.16 μg/$m^{3}$ whereas it was 1.19 μg/$m^{3}$ for NNAR model, 11.47 μg/$m^{3}$ for ARIMA model and 0.70 μg/$m^{3}$ for Hybrid model. In addition, we were able to obtain an acceptable trade-off between accuracy and computation time. The computation time using ESD model was 30 s which is significantly lower than other models. Our model is easy to implement and can be applied to other cities as well. The results can be used by environment protection agencies for policy management as well.

Although we have been able to achieve significant results, there are still some points we would like to address in future work. One task would be to include other weather features such as wind speed and wind direction to further improve the forecast accuracy. In addition, we would like to extend the model to do forecast for longer duration, e.g., 12, 24 and 48 h. The final task would include using the forecasting model to develop a real-time forecasting service in Taiwan.

## Figures and Tables

**Table 1:** Comparative analysis with baseline models.

Models	Mean Error (μg/$m^{3}$)	Computation Time (s)
ESD	0.16	30
ARIMA	11.47	110
NNAR	1.19	410
HYBRID	0.70	600

## Abbreviations

The following abbreviations are used in this manuscript:

PM2.5	Fine particulate matter with diameter less than 2.5 micrometers
IoT	Internet of Things
AQM	Air Quality Management
IoH	Internet of Humans
HCC	Human Centered Computing
MQTT	Message Queuing Telemetry Transport
IDW	Inverse Distance Weighting
ARIMA	Autoregression Integrated Moving Average
ANN	Artificial Neural Network
NNAR	Neural Network Autoregression
ESD	Exponential Smoothing with Drift
MAE	Mean Absolute Error
CDF	Commulative Distribution Function

## References

[1] Hamra G.B., Guha N., Cohen A., Laden F., Raaschou-Nielsen O., Samet J.M., Vineis P., Forastiere F., Saldiva P., Yorifuji T. (2014). Outdoor particulate matter exposure and lung cancer: A systematic review and meta-analysis. Environ. Health Perspect..

[2] Zhao J., Liu C.H., Chen M., Liu X., Leung K.K. Energy-efficient dynamic event detection by participatory sensing. Proceedings of the IEEE International Conference on Communications (ICC).

[3] Yang J., Zhou J., Lv Z., Wei W., Song H. (2015). A real-time monitoring system of industry carbon monoxide based on wireless sensor networks. Sens. J..

[4] Zheng Y., Yi X., Li M., Li R., Shan Z., Chang E., Li T. Forecasting fine-grained air quality based on big data. Proceedings of the 21th ACM SIGKDD International Conference on Knowledge Discovery and Data Mining.

[5] Marchuk G. (2012). Numerical Methods in Weather Prediction.

[6] Dong G.H., Zhang P., Sun B., Zhang L., Chen X., Ma N., Yu F., Guo H., Huang H., Lee Y.L. (2012). Long-term exposure to ambient air pollution and respiratory disease mortality in Shenyang, China: A 12-year population-based retrospective cohort study. Respiration.

[7] Gao Y., Dont W., Guo K., Liu X., Chen Y., Liu X., Bu J., Chen C. Mosaic: A Low-Cost Mobile Sensing System for Urban Air Quality Monitoring. Proceedings of the 35th Annual IEEE International Conference on Computer Communications.

[8] Cheng Y., Li X., Li Z., Jiang S., Li Y., Jia J., Jiang X. AirCloud: A Cloud-based Air-Quality Monitoring System for Everyone. Proceedings of the 12th ACM Conference on Embedded Network Sensor Systems(ACM).

[9] Chen L.J., Ho Y.H., Lee H.C., Wu H.C., Liu H.M., Hsieh H.H., Huang Y.T., Lung S.C.C. (2017). An Open Framework for Participatory PM2.5 Monitoring in Smart Cities. IEEE Access.

[10] Grover A., Kapoor A., Horvitz E. A deep hybrid model for weather forecasting. Proceedings of the 21th ACM SIGKDD International Conference on Knowledge Discovery and Data Mining (ACM).

[11] Izzah A., Sari Y.A., Widyastuti R., Cinderatama T.A. Mobile app for stock prediction using Improved Multiple Linear Regression. Proceedings of the 2017 International Conference on Sustainable Information Engineering and Technology (SIET).

[12] Cortina-Januchs M.G., Quintanilla-Dominguez J., Vega-Corona A., Andina D. (2015). Development of a model for forecasting of PM10 concentrations in Salamanca, Mexico. Atmos. Pollut. Res..

[13] Kitchin R. (2014). The real-time city? Big data and smart urbanism. GeoJournal.

[14] Ghazali R., Hussain A.J., Liatsis P. (2011). Dynamic Ridge Polynomial Neural Network: Forecasting the univariate non-stationary and stationary trading signals. Expert Syst. Appl..

[15] Hsieh H.P., Lin S.D., Zheng Y. Inferring air quality for station location recommendation based on urban big data. Proceedings of the 21th ACM SIGKDD International Conference on Knowledge Discovery and Data Mining.

[16] Khan Z., Anjum A., Kiani S.L. Cloud based big data analytics for smart future cities. Proceedings of the 2013 IEEE/ACM 6th international conference on utility and cloud computing.

[17] Donnelly A., Misstear B., Broderick B. (2015). Real time air quality forecasting using integrated parametric and non-parametric regression techniques. Atmos. Environ..

[18] Zheng Y., Capra L., Wolfson O., Yang H. (2014). Urban computing: Concepts, methodologies, and applications. ACM Trans. Intell. Syst. Technol. (TIST).

[19] Zhu J.Y., Zhang C., Zhang H., Zhi S., Li V.O., Han J., Zheng Y. pg-Causality: Identifying Spatiotemporal Causal Pathways for Air Pollutants with Urban Big Data. https://ieeexplore.ieee.org/abstract/document/7970191/.

[20] Shi X., Li Q., Qi Y., Huang T., Li J. An accident prediction approach based on XGBoost. Proceedings of the 12th International Conference on Intelligent Systems and Knowledge Engineering (ISKE).

[21] Chen T., Guestrin C. Xgboost: A scalable tree boosting system. Proceedings of the 22nd ACM Sigkdd International Conference on Knowledge Discovery and Data Mining.

[22] Chen L.J., Ho Y.H., Hsieh H.H., Huang S.T., Lee H.C., Mahajan S. (2018). ADF: An Anomaly Detection Framework for Large-scale PM2. 5 Sensing Systems. IEEE Internet Things.

[23] Standard O. MQTT version 3.1.1. http://docs.oasis-open.org/mqtt/mqtt/v3.

[24] Mahajan S., Liu H.M., Tsai T.C., Chen L.J. (2018). Improving the Accuracy and Efficiency of PM2.5 Forecast Service Using Cluster-Based Hybrid Neural Network Model. IEEE Access.

[25] Assimakopoulos V., Nikolopoulos K. (2000). The theta model: A decomposition approach to forecasting. Int. J. Forecast..

[26] Hyndman R.J., Billah B. (2003). Unmasking the Theta method. Int. J. Forecasting.

[27] Christodoulos C., Michalakelis C., Varoutas D. (2010). Forecasting with limited data: Combining ARIMA and diffusion models. Technol. Forecast. Soc..

[28] Da Veiga C.P., Da Veiga C.R.P., Catapan A., Tortato U., Da Silva W.V. (2014). Demand forecasting in food retail: A comparison between the Holt-Winters and ARIMA models. WSEAS Trans. Bus. Econ..

[29] Hyndman R.J., Athanasopoulos G. Forecasting: Principles and practice. https://books.google.com.hk/books?hl=en&lr=&id=_bBhDwAAQBAJ&oi=fnd&pg=PA7&dq=Forecasting:+principles+and+practice&ots=Thh0wn0NNM&sig=q9LrK5cjJNR0PGWdJguSy_QH91M&redir_esc=y#v=onepage&q=Forecasting.

